# Neoadjuvant rituximab modulates the tumor immune environment in patients with high risk prostate cancer

**DOI:** 10.1186/s12967-020-02370-4

**Published:** 2020-05-28

**Authors:** Stephen T. Ryan, Jing Zhang, Danielle N. Burner, Michael Liss, Emily Pittman, Michelle Muldong, Ahmed Shabaik, Jason Woo, Nicole Basler, Jonathan Cunha, Shabnam Shalapour, Monica V. Estrada, Michael Karin, Karen Messer, Stephen Howell, Christopher J. Kane, Christina A. M. Jamieson

**Affiliations:** 1 Department of Urology, UCSD Moores Cancer Center, University of California San Diego School of Medicine, 3855 Health Sciences Drive, Mail Code: 0987, La Jolla, CA 92093-0987 USA; 2 Department of Pathology, University of California San Diego School of Medicine, La Jolla, CA USA; 3 Department of Pharmacology, University of California San Diego School of Medicine, La Jolla, CA USA; 4 Department of Pharmacology, University of California San Diego School of Medicine, La Jolla, CA USA; 5 Department of Medicine, University of California San Diego School of Medicine, La Jolla, CA USA; 6 Division of Biostatistics, Department of Family and Preventive Medicine, University of California San Diego School of Medicine, La Jolla, CA USA; 7 Department of Urology, University of Texas Health Science Center San Antonio, San Antonio, TX USA; 8 Biorepository and Tissue Technology Shared Resource at the University of California San Diego School of Medicine, La Jolla, CA USA

**Keywords:** Prostate cancer, Rituximab, Immunotherapy, CD20, CD3, PD-L1, Neoadjuvant, Prostatectomy, Tumor infiltrating lymphocytes (TILs)

## Abstract

**Background:**

Immunotherapeutic regulation of the tumor microenvironment in prostate cancer patients is not understood. Most antibody immunotherapies have not succeeded in prostate cancer. We showed previously that high-risk PCa patients have a higher density of tumor infiltrating B-cells in prostatectomy specimens. In mouse models, anti-CD20 antibody ablation of B-cells delayed PCa regrowth post-treatment. We sought to determine whether neoadjuvant anti-CD20 immunotherapy with rituximab could reduce CD20+ B cell infiltration of prostate tumors in patients.

**Methods:**

An open label, single arm clinical trial enrolled eight high-risk PCa patients to receive one cycle of neoadjuvant rituximab prior to prostatectomy. Eleven clinical specimens with similar characteristics were selected as controls. Treated and control samples were concurrently stained for CD20 and digitally scanned in a blinded fashion. A new method of digital image quantification of lymphocytes was applied to prostatectomy sections of treated and control cases. CD20 density was quantified by a deconvolution algorithm in pathologist-marked tumor and adjacent regions. Statistical significance was assessed by one sided Welch’s t-test, at 0.05 level using a gatekeeper strategy. Secondary outcomes included CD3+ T-cell and PD-L1 densities.

**Results:**

Mean CD20 density in the tumor regions of the treated group was significantly lower than the control group (p = 0.02). Mean CD3 density in the tumors was significantly decreased in the treated group (p = 0.01). CD20, CD3 and PD-L1 staining primarily occurred in tertiary lymphoid structures (TLS). Neoadjuvant rituximab was well-tolerated and decreased B-cell and T-cell density within high-risk PCa tumors compared to controls.

**Conclusions:**

This is the first study to treat patients prior to surgical prostate removal with an immunotherapy that targets B-cells. Rituximab treatment reduced tumor infiltrating B and T-cell density especially in TLSs, thus, demonstrating inter-dependence between B- and T-cells in prostate cancer and that Rituximab can modify the immune environment in prostate tumors. Future studies will determine who may benefit from using rituximab to improve their immune response against prostate cancer.

*Trial registration* NCT01804712, March 5th, 2013 https://clinicaltrials.gov/ct2/show/NCT01804712?cond=NCT01804712&draw=2&rank=1

## Background

The tumor microenvironment plays a role in cancer cell proliferation, immune evasion, metastasis, and treatment resistance which is mediated by direct cancer cell contact and/or indirect cell signaling through cytokines, chemokines, and growth factors [1, 2]. Successful targeting of T-cell immune checkpoint pathways has shown dramatic responses in this environment for multiple malignancies but has failed as a monotherapy in prostate cancer (PCa) [3–9]. This may be due to the unique nature of the PCa tumor immune microenvironment [10]. In PCa mouse models, the presence of an immunosuppressive B-cell subpopulation was associated with accelerated recurrence of castrate resistant PCa [11] and suppressed the cytotoxic T-cell response normally associated with chemotherapy [12]. In prostatectomy specimens, we have demonstrated previously that high B-cell density is associated with biochemical failure in high-risk patients [11, 13]. While inhibition of B-cells may provide an alternative therapeutic approach, no clinical trials have attempted to modulate B-cells in the tumor microenvironment.

Rituximab is a well-tolerated monoclonal antibody against the CD20 antigen which is highly expressed on most B-cells. Rituximab was originally approved by the FDA for the treatment of Non-Hodgkin’s Lymphoma, with expanded indications to many non-malignant B-cell related diseases. In a PCa mouse model, anti-CD20 treatment decreased the number of tumor infiltrating B-cells and delayed the onset of castrate resistant disease [11]. We sought to determine whether neoadjuvant treatment of high-risk PCa patients with the anti-CD20 immunotherapy, Rituximab, could reduce B-cell infiltration of PCa tumors.

Men with high-risk PCa were prospectively enrolled to undergo a single 1-month cycle of neoadjuvant rituximab prior to curative intent prostatectomy (NCT01804712). The primary objective was to determine the histological response of tumor infiltrating B-cells. Secondary objectives included assessing the impact on oncological outcomes, serum PSA response, immunohistochemical staining profiles of other immune cells, and observe patient safety and tolerability.

## Methods

### Study design and data sources

#### Study population

After IRB approval, we conducted an open-label non-randomized single arm study of neoadjuvant rituximab in patients with high-risk PCa who were candidates for radical prostatectomy (“PROTUX” NCT01804712). Eight patients were enrolled who presented to a single tertiary care institution and prostatectomies were performed by a high-volume surgeon. High-risk was defined as either Kattan nomogram probability of 5-year disease free status < 60% or a Gleason score ≥ 8 [14]. Inclusion criteria were the ability to understand and provide consent, candidate for curative intent prostatectomy, Eastern Cooperative Oncology Group (ECOG) performance status 0–1, adequate organ function within 21 days of study entry based on routine screening hematologic and biochemical laboratory values. Exclusion criteria included prior treatment for prostatic adenocarcinoma (excluding transurethral resection of prostate), evidence of metastatic disease on cross sectional imaging or bone scan, history of chronic infection (especially hepatitis B or C, or HIV), positive hepatitis B or C serology, concurrent or past use of investigational agents within 1 month of study entry and use of erectile dysfunction medication within 14 days of entry.

#### Study treatment

Enrolled patients received rituximab 375 mg/m^2^ intravenously once weekly for 4 treatments over 28 days. Patients were pre-medicated with diphenhydramine 25 mg intravenously and acetaminophen 650 mg orally. To reduce the risk of infusion reaction, the initial rate was 50 mg/h and was increased by 50 mg/h increments every 30 min if there was no reaction (max = 400 mg/h). Protocol guidelines allowed for dose modification for minor reactions and discontinued infusion for severe reactions. Vital signs were recorded before and after each treatment, laboratory tests were performed at each treatment, along with any medication changes. Prostatectomy was performed within 14 days of last treatment with planned lymph node dissection. Follow-up was conducted for 3 years as standard of care. PSA measurements occurred every 3 months for the first 2 years and every 6 months for the third year. Adverse events were assessed at each study visit for the duration of study and for 28 days after the discontinuation of rituximab.

### Procedures

#### Tissue procurement

Whole-mount resected prostate tissue was processed according to standard institutional procedures. Routine histological assessment for tumor staging was performed and then excess tissue was made available for research purposes. The control group consisted of archived radical prostatectomy specimens stored in a clinical pathology lab, selected for similar pathologic characteristics. Slides were prepared in blinded fashion from eight treated prostatectomies (two slides from one multifocal specimen) and 11 controls. Samples were labeled with the subject’s de-identified study number and collection date, and all of the following processing was performed concurrently.

#### Immunohistochemistry

Formalin fixed, paraffin embedded prostatectomy tissue blocks with the highest percentage of tumor were selected by clinical urologic pathologist (AS) and 4 µm thick serial sections were labeled with anonymized slide labels for blinded immunohistochemical staining and analysis. First and last serial sections were stained with hematoxylin and eosin and pathology was confirmed by the pathologist. Staining was performed as previously described with minor modifications [13]. Immunohistochemical staining was performed by the UCSD Moores Cancer Center Tissue Technology Shared Resources manually for anti-CD20, catalog # MO755 (Agilent Dako) then on IntelliPath (Biocare Medical) Autostainer using anti-CD3 catalog # CME324A (Biocare Medical) and anti-PD-L1 catalog #13684S (Cell Signaling), HRP polymers from Biocare Medical, RHRP520 L and RT517 L labeled Envision anti-mouse DAKO catalog # K4000 and DAB substrate. The ChromPure Rabbit IgG catalog # 011-000-003 (Jackson Laboratories) isotype control antibody was used to determine background staining threshold. Tumor regions on stained slides were marked by a blinded pathologist (AS).

#### Histological response/digital microscope image scanning

All slides were digitally scanned at 40× magnification at Tissue Technology Shared Resources using the Aperio AT2 system (Leica BioSystems), the background illumination levels were calibrated using an automated pre-scan procedure. The acquired digital images representing whole tissue sections were evaluated for image quality and rescanned if needed. Slide images were viewed and analyzed using ImageScope viewer. Immunohistochemical staining was quantified by computer algorithm in tumor and adjacent tissue regions using an Aperio™ Digital Pathology Slide Scanner as previously described [13]. The Spectrum Analysis algorithm package and ImageScope analysis software were applied to quantify IHC staining. The algorithm used was the “color deconvolution” (version 9; Aperio Technologies, Inc.). Color deconvolution algorithm was applied to each stained section, tested on negative and positive control slides and tuned on specimen slides. Positive staining thresholds for low, intermediate and high intensity staining and threshold for background staining were set using anti-CD20, anti-CD3 or anti-PD-L1 on positive control human spleen sections and isotype-matched negative control antibody stained sections.

#### Histological response/digital microscope image analysis

ImageScope pen tool was used to outline the pathologist’s marked tumor region on each digital slide image and analysis was performed using color deconvolution algorithm as previously described [13]. The negative pen tool was used to exclude artifacts within an analysis area (ex: folds, tears, large clear areas devoid of tissue, and non-cellular precipitates of concentrated stain). The stained area (mm^2^) and total area (mm^2^) were calculated within the tumor region. The same was performed for all specimen on the slide (tumor plus normal adjacent tissue regions) and normal adjacent tissue areas were determined by simple subtraction (All specimen–tumor region). The ratio of total stained area over total area (mm^2^/mm^2^) was determined for both tumor region and normal adjacent tissue.

### Statistical analysis

#### Study design and outcomes

The original primary endpoint was the rate of histologic response, defined as having B-cell density within the tumor below a pre-determined threshold (18th percentile), comparing to concurrently assayed control samples. Using a Simon’s two stage design at 5% significance with 80% power to detect a response rate of 50% or more against a null hypothesis of 20% response, the original sample size was estimated to include 18 treated and 27 controls specimens. However, the trial was closed due to low accrual after 8 treated subjects, and the primary endpoint was revised prior to analysis. Study personnel were blinded during the outcome assessment. The dual primary hypotheses were (1) mean CD20 density in tumor would be less in treated specimens compared to controls; and (2) the mean within-patient difference, tumor minus adjacent non-neoplastic tissue, would be less in treated compared to control specimens. Primary hypotheses were tested at overall 5% level using a one-sided Welch’s t-test, using a gatekeeper strategy to correct for the 2 comparisons. Secondary endpoints included CD3 and PDL-1 density, biochemical recurrence, change in serum PSA, serum leukocyte count, and adverse event rates.

## Results

Eight men were enrolled and treated with neoadjuvant rituximab and compared to 11 controls. Mean age of the treatment and control patients was 62.3 (± 5.2) and 63.4 (± 6.4) years, respectively. Both groups were mostly Caucasian (87.5% and 90.9%) and non-Hispanic (75% and 81.8%). PSA at diagnosis was 7.7 (± 4.7) and 7.5 (± 4.7) ng/mL for the treated and control groups, respectively. The pathologic stage of treated specimens were 37.5% pT2a-c and 62.5% pT3a-b, with Gleason Grade risk groups II (n = 1, 12.5%), III (n = 2, 25%), and IV&V (n = 5, 62.5%). The control prostatectomies consisted of 18.2% pT2a-c and 81.8% pT3a-b, and Gleason Grade risk groups I (n = 3, 27.3%), and IV and V (n = 8, 72.7%). Note, all Gleason risk group I (GS 3 + 3) prostatectomies in the control group were pT3a. Descriptive characteristics are listed in Table 1.

**Table 1:** Demographics

	Rituximab (n = 8)	Controls (n = 11)
Age (mean/SD)	62.3 (5.2)	63.4 (6.4)
Race (%)		
White	7 (87.5)	10 (90.9)
Unknown	1 (12.5)	1 (9.1)
Ethnicity (%)		
Hispanic	1 (12.5)	1 (9.1)
Non-hispanic	6 (75)	9 (81.8)
Unknown	1 (12.5)	1 (9.1)
PSA at diagnosis (mean/SD)	7.7 (4.7)	7.5 (4.7)
Gleason risk group after prostatectomy (n/%)		
I (GS 3 + 3)	–	3 (27.3)^a^
II (GS 3 + 4)	1 (12.5)	–
III (GS 4 + 3)	2 (25)	–
IV and V (GS ≥ 8)	5 (62.5)	8 (72.7)
pT stage		
pT2a-c	3 (37.5)	2 (18.2)
pT3a-b	5 (62.5)	9 (81.8)

^a^All were pT3a

### Primary outcomes

**Fig. 1:**
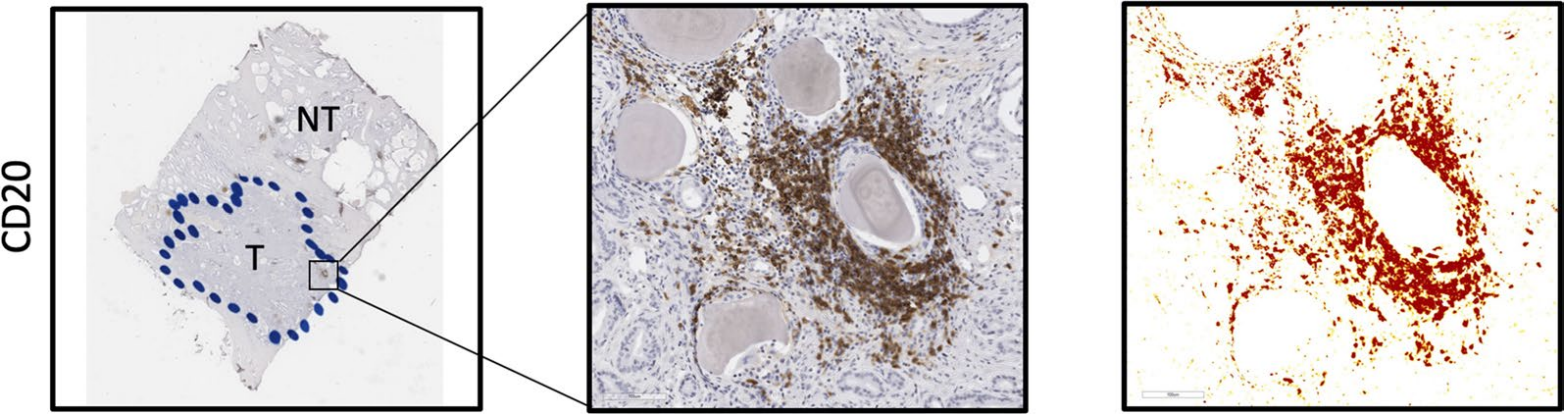
Immunohistochemical staining of serial prostatectomy sections showed presence of CD20+ B-cells cells aggregated in immune cell foci known as tertiary lymphoid structures (TLSs). **a** Representative AperioScope scanned image of anti-CD20 stained prostatectomy section counter-stained with hematoxylin. Tumor regions outlined by pathologist markings in blue, T = tumor, NT = non-tumor, ×10 magnification. **b** Box inset enlarged at ×200 magnification shows CD20+ B-cells stained brown in bright-field. **c** Post-deconvolution image of CD20 staining. After Imagescope deconvolution algorithm is run the stained color intensity is represented as image pixels with high intensity (brown), intermediate (orange) and low (yellow) staining intensity

**Fig. 2:**
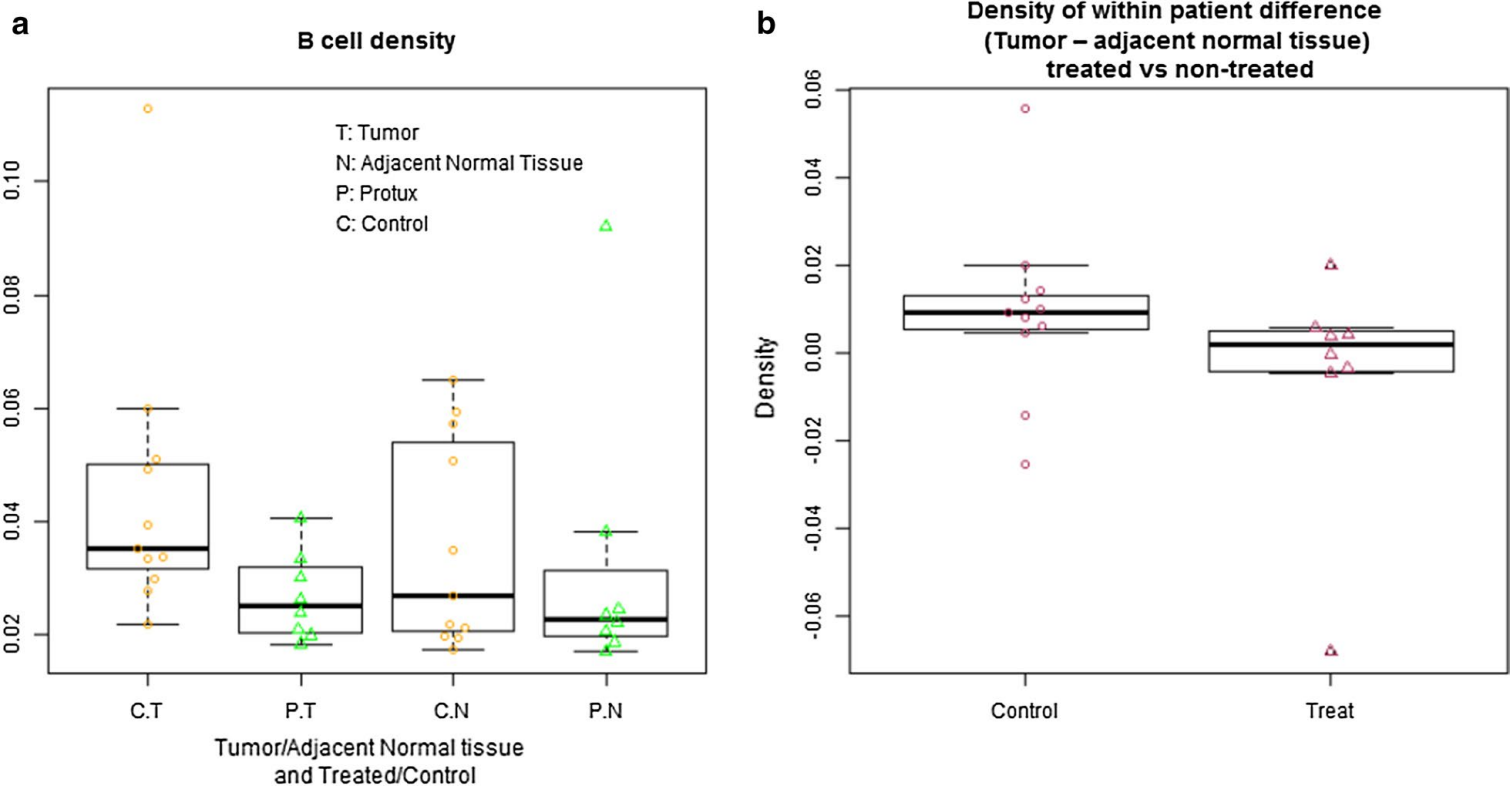
Primary outcome analysis of B cell density in prostatectomy tissue showed decrease in B-cell density in tumor regions after neoadjuvant Rituximab treatment. **a** Boxplot and scatter plot for B cell density (Treated vs. Control), B-cell density in treated: 0.027 mm^2^/mm^2^ (95% CI 0.021, 0.033) and 0.044 mm^2^/mm^2^ (95% CI 0.028, 0.062) in the controls, p = 0.02 for Welch two sample t-test comparing B-cell density of Tumor in treated group and historic control group, i.e. The mean B cell density in tumor was significantly lower in treated subjects compared to historical control subjects. C = control samples, P = PROTUX Rituximab-treated, T = marked tumor region, N = adjacent normal tissue. **b** Within subject difference of B-cell density: tumor minus adjacent normal tissue (Treated vs. Controls)

Immunohistochemical analysis of the B-cell marker, CD20, was performed on radical prostatectomy tissue as shown in Fig. 1. Immunohistochemical staining of serial prostatectomy sections showed the presence of CD20+ B-cells cells aggregated in immune cell foci known as tertiary lymphoid structures (TLS). B-cell density within the tumor region of the rituximab treated samples was 0.027 mm^2^/mm^2^ (95% CI 0.021, 0.033) and 0.044 mm^2^/mm^2^ (95% CI 0.028, 0.062) in the controls (Fig. 2a). A significant difference in B-cell density was detected (p = 0.02) (Table 2). The non-neoplastic adjacent tissue had a B-cell density of 0.032 mm^2^/mm^2^ in the treated prostatectomies and 0.036 mm^2^/mm^2^ in the control specimens, with no difference observed (p = 0.36) (Fig. 2a, Table 2). The mean within group CD20+ difference (tumor − non-neoplastic adjacent densities) was − 0.005 mm^2^/mm^2^ (95% CI − 0.028, 0.017) in the treated and 0.009 mm^2^/mm^2^ (95% CI − 0.004, 0.023) in the control groups, suggesting a greater overall CD20+ decrease in the treated tissue, however, it did not reach, statistical significance (p = 0.11) (Fig. 2b, Table 2). All p-values from one-sided Welch’s t-tests.

**Table 2:** Primary analysis: results

B-cell density (mm^2^/mm^2^)	Rituximab Mean (95% CI)	Controls Mean (95% CI)	*p*
Tumor region	0.027 (0.021, 0.033)	0.044 (0.028, 0.062)	0.02
Non-neoplastic region	0.032 (0.011, 0.053)	0.036 (0.023, 0.048)	0.36
Within group difference			
Tumor − non-neoplastic	− 0.005 (− 0.028, + 0.017)	0.009 (− 0.004, + 0.023)	0.11

Mean B-cell densities (95% CI). (Top) B cell density within the Tumor and Normal tissue regions. (Bottom) Comparison of within group difference

1. Density of Tumor for subjects treated with Rituximab

2. Density of adjacent normal tissue for subjects treated with Rituximab

3. Use one sample t test to get the mean and 95% CI. p value is from two sample t-test

For two sample t-test, the Welch (or Satterthwaite) approximation to the degrees of freedom is used

### Secondary outcomes

**Fig. 3:**
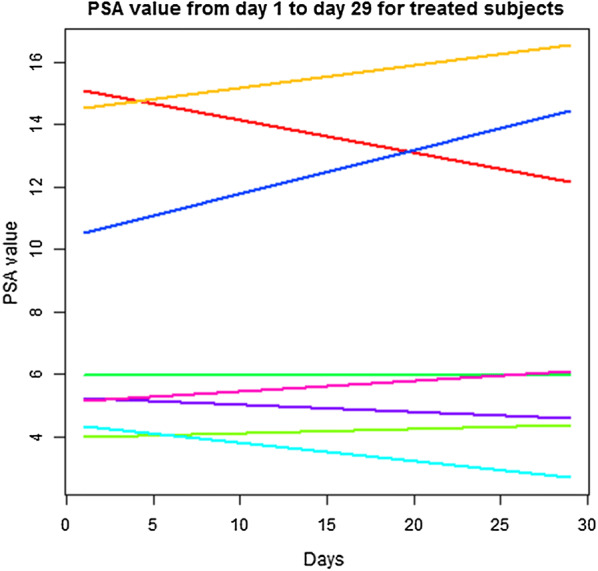
Secondary outcome analysis: serum PSA from Day 1 to Day 29 not significantly changed in Rituximab treated patients. Serum PSA did not appreciably change over the course of neoadjuvant rituximab (Day1: 8.1 ng/mL (± 4.3), Day 29: 8.36 ng/mL (± 4.88), p = 0.84)

Absolute lymphocyte count decreased by − 0.29 [1000/mm^3^] (95% CI − 0.7, 0.12). No patients experienced leukopenia or thrombocytopenia throughout the study period. Serum PSA did not change appreciably over the course of neoadjuvant rituximab (Day1: 8.1 ng/mL (95% CI 4.26, 11.94), Day 29: 8.36 ng/mL (95% CI 4.00, 12.72), p = 0.84, Wilcoxon signed rank test) (Table 3 and Fig. 3). For the 14 patients with 6-month follow-up, 2 patients in the control group and 1 patient in the treated group had a persistently elevated PSA. Four patients in the control group did not have 6-month follow-up; three were lost to follow-up, and the other died of unrelated causes. One patient in the treated group was lost to follow-up.

**Table 3:** Secondary analysis 1—change in PSA pre-treatment to day 29

Patient study ID	Early PSA	Day 29 PSA
1	15.08	12.14
2	14.5	16.52
3	3.98	4.4
4	5.98	5.96
5	4.32	2.7
6	10.52	14.43
7	5.26	4.61
8	5.16	6.09

To determine the effectiveness of neoadjuvant rituximab in the treatment of prostate cancer as evaluated by the serum PSA

Wilcoxon signed rank test, p-value = 0.84

**Fig. 4:**
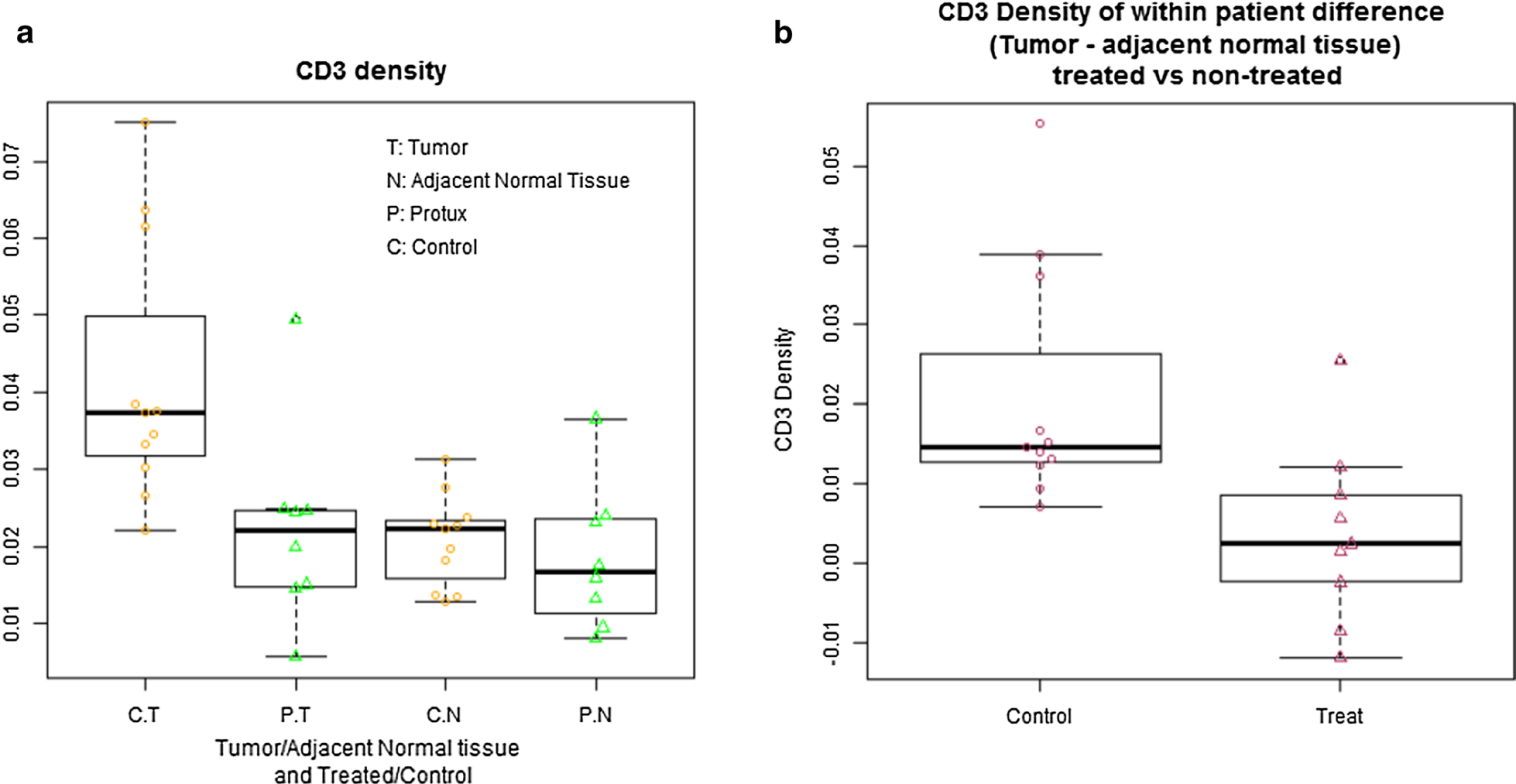
Secondary analysis showed decrease in CD3 + T-cell density. **a** CD3 plot Boxplot and scatter plot for CD3 density (Treated vs. Control), p = 0.995 for Welch two sample t-test comparing CD3+ T-cell density of Tumor in treated group and historic control group, i.e. the mean CD3+ T-cell density in tumor was significantly lower in treated subjects compared to historical control subjects. Mean CD3+ T-cell density in the tumor region of the Rituximab-treated group was 0.0223 mm^2^/mm^2^ (95% CI 0.0116, 0.033) and 0.0419 mm^2^/mm^2^ (95% CI 0.0305, 0.0533) in the control group. **b** Within subject difference of CD3 density: tumor minus adjacent normal tissue (Treated vs. Controls)

Immunohistochemical staining of serial prostatectomy sections showed the presence of CD20+ B-cells, CD3+ T-cells and PD-L+ cells aggregated in immune cell foci known as tertiary lymphoid structures (TLS) in tumor and non-tumor regions (Additional file 1: Figures S1, S2). Density was calculated for the pan T-cell marker, CD3, and the immune checkpoint marker, PD-L1 (Fig. 1). The mean CD3+ T-cell density in the tumor region of the rituximab-treated group was 0.022 mm^2^/mm^2^ (95% CI 0.012, 0.033) and 0.042 mm^2^/mm^2^ (95% CI 0.031, 0.053) in the control group. A significant decrease in CD3+ T cell density was detected (p = 0.01) (Fig. 4a, Table 4). The mean within group CD3+ difference (tumor − normal adjacent densities) was 0.004 mm^2^/mm^2^ (95% CI − 0.005, 0.013) in the treated and (tumor − normal adjacent densities) was 0.021 mm^2^/mm^2^ (95% CI 0.011, 0.031) in the control groups, suggesting a greater overall CD3 + decrease in the treated tissue which did reach statistical significance (p = 0.01) (Fig. 4b, Table 4). All p-values except serum PSA from two-sided Welch’s t-tests.

**Table 4:** Secondary analysis: CD3, a pan T-cell marker

CD3+ density (mm^2^/mm^2^)	Rituximab Mean (95% CI)	Controls Mean (95% CI)	*p*
Tumor region	0.022 (0.012, 0.033)	0.042 (0.031, 0.053)	0.01
Non-neoplastic region	0.019 (0.011, 0.026)	0.021 (0.017, 0.025)	0.56
Within group difference			
Tumor − non-neoplastic	0.004 (− 0.005, + 0.013)	0.021 (+ 0.011, + 0.031)	0.01

1. Density of tumor for subjects treated with Rituximab

2. Density of adjacent normal tissue for subjects treated with Rituximab

3. Use one sample t test to get the mean and 95% CI

4. p value is from two sample t-test

For two sample t-test, the Welch (or Satterthwaite) approximation to the degrees of freedom is used

Null hypothesis is no difference. Alternative hypothesis is true difference in means is greater than 0

5. (Tumor − Normal) is calculated by: column D − column E

**Fig. 5:**
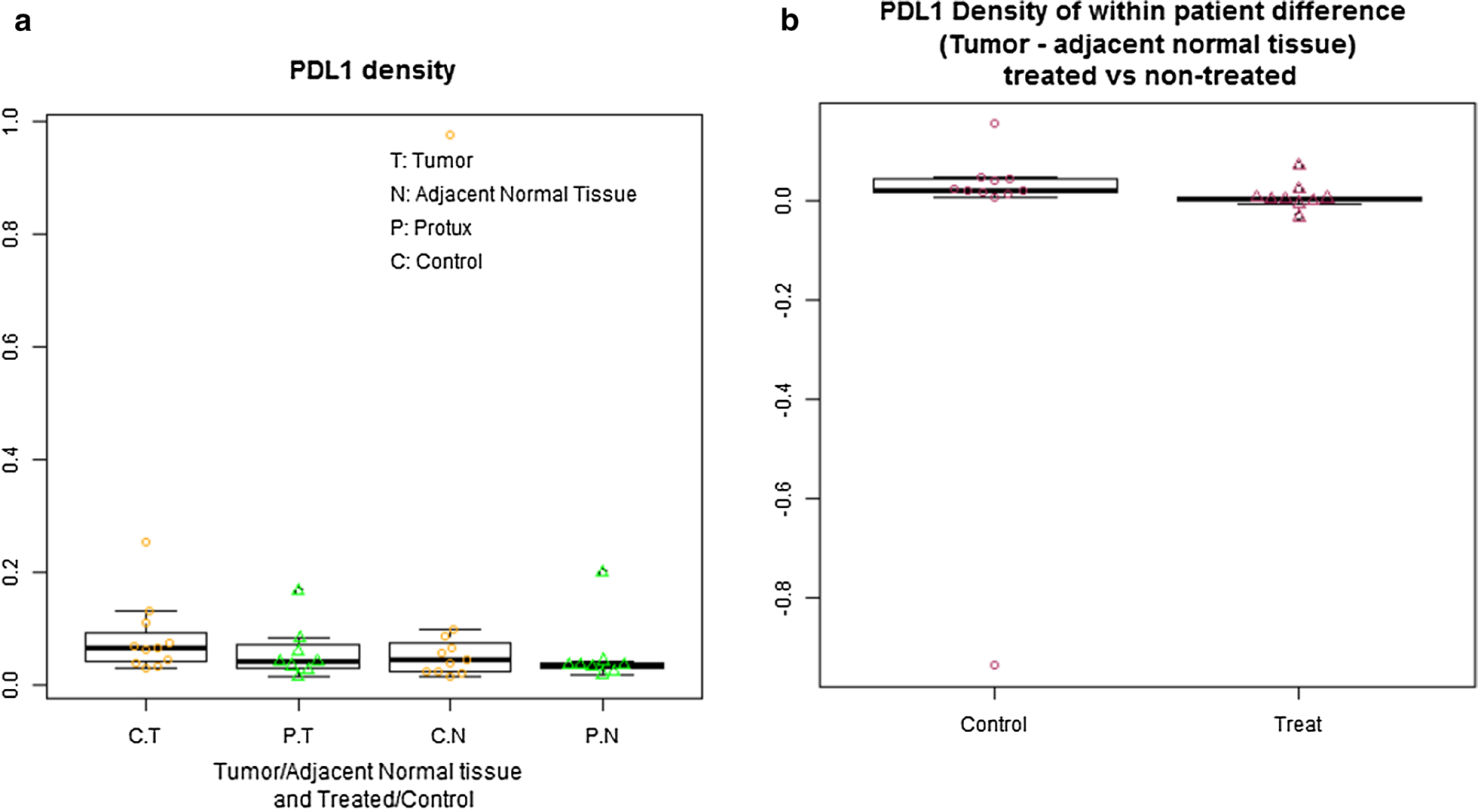
Secondary analysis of PD-L1 immunohistochemical staining density. **a** Boxplot and scatter plot for PD-L1 density (Treated vs Control), mean PD-L1 density in the treated (0.0589) versus control samples (0.0833) was observed but was not significant: p = 0.245 for Welch two sample t-test comparing PD-L1+ cell density of tumor in treated group and historic control group, i.e. the mean PD-L1+ cell density in tumor was significantly lower in treated subjects compared to historical control subjects. **b** Within subject difference of PDL1 density: tumor minus adjacent normal tissue (Treated vs. Controls)

A non-significant decrease in the mean PD-L1 density (mm^2^/mm^2^) in the treated (0.059) versus control samples (0.083) was observed (p = 0.36) (Table 5, Fig. 5a). The mean within group PD-L1+ difference (tumor − normal adjacent densities) was 0.005 mm^2^/mm^2^ (95% CI (− 0.012, 0.022) in the treated and − 0.049 mm^2^/mm^2^ (95% CI − 0.248, 0.151) in the control groups, suggesting a greater overall PD-L1+ decrease in the treated tissue; however, it did not reach statistical significance (p = 0.56) (Fig. 5b, Table 5).

**Table 5:** Secondary analysis: PD-L1

PD-L1 density (mm^2^/mm^2^)	Rituximab Mean (95% CI)	Controls Mean (95% CI)	*p*
Tumor region	0.059 (0.018, 0.099)	0.083 (0.040, 0.127)	0.36
Non-neoplastic region	0.054 (0.004, 0.103)	0.132 (0.056, 0.321)	0.39
Within group difference			
Tumor − non-neoplastic	0.005 (− 0.012, + 0.022)	− 0.049 (− 0.248, + 0.151)	0.56

1. Density of tumor for subjects treated with rituximab

2. Density of adjacent normal tissue for subjects treated with rituximab

3. Use one sample t test to get the mean and 95% CI

4. p value is from two sample t-test

For two sample t-test, the Welch (or Satterthwaite) approximation to the degrees of freedom is used

Null hypothesis is no difference

5. (Tumor − normal) is calculated by: column K − column L

### Adverse event

All enrolled patients were able to complete rituximab treatment. Four patients experienced a total of 10 adverse events, 9/10 were Grade 1 and 2, with 4 of those 9 being possible or probably related to rituximab. There was only one event considered severe, a grade 4 thromboembolic event which occurred after surgery, deemed unrelated to rituximab. The patient was treated with anticoagulation without incident. No adverse events were definitively related to rituximab. Two patients experienced an infusion related reaction (Grade 2) after the initial dose. Both resolved and tolerated dose escalation per protocol. Results are listed in Table 6.

**Table 6:** Secondary analysis 4: safety and tolerability of neo-adjuvant rituximab

Severity/grade	Adverse events (n)	Possible, probable, or definite attribution (n)	Description of related adverse events (n of patients affected)
Mild/1	6	2	Fatigue (×2)
Moderate/2	3	2	Infusion related reaction (×2)
Severe/3	0	0	
Life-threatening/4	1	0	
Fatal/5	0	0	
Total	10	4	

Adverse events (description, timing, grade [CTCAE v4.03], severity, seriousness, and relatedness)

## Discussion

Immunoregulation of the tumor microenvironment in human prostate cancer is not well understood [1, 2, 15]. In the present study, we demonstrated that neoadjuvant rituximab significantly decreased B-cell density within tumors compared to concurrently assayed controls (p = 0.02) and appeared to reduce the density of tumor resident B-cells to levels comparable to adjacent non-neoplastic tissue, (p = 0.11 relative to controls). There was also a significant decrease in CD3+ T-cell density within the tumor regions demonstrating the inter-dependence between B and T-cells in prostate cancer. Neoadjuvant rituximab was well tolerated with no attributable serious adverse events. Therefore, these results provide evidence that rituximab is a safe modifier of the immune environment within prostate tumors.

Lymphocyte infiltration plays a central role in cancer cell apoptosis and has been linked to tumor stage and recurrence free survival [2, 10, 14, 16]. Observational studies of B-cell density in PCa have had conflicting results, with some reporting lower B-cells counts [17], while others described higher B-cell density only in high-grade tumors [18]. The prognostic implications of B-cell density in PCa are also mixed. Petitprez and co-workers [19] showed recently that a high density of CD8+ cells plus PD-L1+ cells was associated with higher risk of biochemical recurrence in lymph node-positive patients, but no association was found with CD20+ cells.

Our prior work has shown increased tumor infiltrating CD20+ B-cells were correlated with biochemical recurrence [13], while others remark that B-cells have no association with biochemical recurrence [20, 21]. All studies agree that B-cells are rare in PCa tumors, making the variations in results likely due to differences in analysis methods and lymphocyte quantification. To our knowledge this is the first trial to prospectively modulate B-cell density within PCa tumors. Pre-clinical work in PCa mouse models demonstrated a B-cell driven pathway that promoted castration resistance [11, 12]. Furthermore, decreased or depleted B-cell numbers within the tumor were associated with inhibited tumor growth and delay of metastasis. The current study was a successful phase I translation of the mouse model, whereby rituximab was able to decrease B-cell density in the PCa tumor.

The majority of immunotherapies are based on augmenting CD8+ cytotoxic T-cells (CTL), which normally assist in eradicating tumor cells but can become quiescent over time [22, 23]. Observational studies of PCa found that rare spontaneous regressions were associated with the lack of CD4+ T-regulatory cells while progression was associated with increased CD4+ T-regulatory cells, with no significant differences in CD4+ T-helper or CD8+ T-cytotoxic concentrations [24, 25]. In animal models of advanced PCa, B-cell interactions were responsible for down-regulating T-cell dependent cytotoxicity, while B-cell depletion had no effect on the number of CD4+ Treg cells within the tumor [12]. Following this result, we expected decreasing B-cells would have no effect on the number of T-cells in the treatment arm, and thus the decreased CD3+ density, a pan T-cell marker, came as a surprise. To verify our finding, all samples were re-analyzed by three blinded researchers, with the CD3+ density staying within 5% of the original findings. In different patient populations, rituximab has shown a range of T cell responses from no change in T-cell concentration [24], to an increase in all T-cell subpopulations [26], or dramatic decrease in CD4+ Treg cells [27]. These studies highlight that enumerating lymphocytes is often not sufficiently informative about functional immune interactions. The clinical implications of our findings are as yet unknown, as PCa emergence may be related to unique inhibitory pathways [28]. Future studies will concentrate on defining specific lymphocyte and monocyte subpopulations and associated signaling and to better understand B and T cell interactions in prostate tumor regions compared to benign regions.

Rituximab was chosen given its efficacy and safety profile. Initially approved in 1997 for the treatment of relapsed or refractory B-cell Non-Hodgkin’s Lymphoma (NHL), rituximab causes rapid depletion of circulating and tissue-based B-cells generally within the first three doses, with sustained depletion for up to 6–9 months after treatment [29]. Rituximab has subsequently been approved for several additional indications including chronic lymphocytic leukemia (CLL), rheumatoid arthritis, Wegener’s granulomatosis and microscopic polyangiitis. Common adverse reactions observed in clinical trials of lymphoid malignancies were infusion reactions, fever, lymphopenia, neutropenia, chills, infection and asthenia (≥ 25%) [30]. We noted four Grade 1 and 2 adverse events that were probably related to rituximab (infusion reaction and fatigue) occurring in three of the eight patients. Both infusion related reactions were able to be dose increased and complete the full cycle. This suggest a well-tolerated profile in the prostate cancer population.

The strengths of this study are the prospective design with rigorous, blinded assessment of B-cell density in tumor and adjacent tissue. Since the study started, two case reports have described treatment of metastatic PCa patients with rituximab. The first was a 66-year-old male who developed metastatic PCa; a lesion biopsy disclosed prominent CD20+ nests. He was treated with rituximab which resulted in a significant PSA decline [31]. The second was a 79-year-old metastatic PCa patient with a concurrent B-cell lymphoma. On treating his lymphoma with rituximab, he had a dramatic decline in PSA and his skeletal lesions showed signs of resolving [32]. To our knowledge, other than these reports, no other prospective studies of B-cell densities have been conducted.

This study adds to the rapidly evolving field of immunomodulating the tumor microenvironment of PCa. Other studies assessing are correlative retrospective tissue assessment [24, 25] or are evaluations of circulating lymphoid cells [33].

The current study is one of the few neoadjuvant immune trials to report results. Prior neoadjuvant studies either terminated or withdrew (NCT00577356/NCT01197209) or have not published results. Gao et al. [28] demonstrated that neoadjuvant ipilimumab increased CD4+ and CD8+ cells within prostatectomy specimens, but did not mention effects on CD20+ cells.

Weaknesses include unknown impact on clinical outcomes, or impact of T-cell modulation. However, the primary outcome of the study was met, and the study design was not powered for secondary outcomes. Because of the small number of patients in this study it is hard to generalize our results, however this is the only prospective assessment of B-cell modulation in prostate cancer. Our method of lymphocyte annotation is not widely used, but we have published experience using this imaging quantification method for B cells [13]. An important difference between the animal models that served as the background work for the trial design was that they used castrated PCa mouse models, whereas this patient cohort did not receive androgen deprivation therapy.

Recently, three studies showed that the presence of B cells in tumor regions in patients with metastatic melanoma or sarcoma who were treated with immune check-point blockade (ICB) immunotherapy were predictive of good prognosis [34–36]. In particular, B cells that were in tertiary lymphoid structures (TLS) which contain aggregates of multiple immune cell types, were present in responding patients. B cells, therefore, have both positive and negative roles in tumor immunology, and thus, it is critical to elucidate the precise tumor infiltrating B cell and T cell states, or types, and their interactions within TLSs not only for prognosis but also to tailor immunotherapy regimens and improve the anti-tumor response in prostate cancer.

## Conclusions

Neoadjuvant rituximab was well-tolerated and decreased B-cell density within high risk PCa tumors compared to controls. Rituximab appeared to reduce the density of tumor-resident B-cells to levels comparable to adjacent normal tissue. Somewhat unexpectedly, there was a significant decrease in CD3+ T cell density in prostate tumor regions of the rituximab-treated patients, demonstrating the essential interaction of these cell types in prostate cancer. These results provide evidence that rituximab can modify the immune microenvironment of the tumor.

## Supplementary information

**Additional file 1: Figure S1.** Immunohistochemical staining of serial prostatectomy sections showed presence of CD20+ B-cells, CD3+ T-cells and PD-L1+ cells aggregated in immune cell foci known as tertiary lymphoid structures (TLS) in tumor region. (A) Representative AperioScope scanned image of anti-CD20 stained prostatectomy section counter-stained with hematoxylin. Tumor regions outlined by pathologist markings in blue, T = tumor, NT = non-tumor, 10X magnification. (B) Box inset enlarged at 200× magnification shows CD20+ B-cells stained brown in bright-field. (C) Post-deconvolution image of CD20 staining. After Imagescope deconvolution algorithm is run the stained color intensity is represented as image pixels with high intensity (brown), intermediate (orange) and low (yellow) staining intensity. Digital images of serial prostatectomy sections and de-convoluted images stained with anti-CD3 (D, E) and anti-PD-L1 (F, G). **Figure S2.** Immunohistochemical staining of serial prostatectomy sections showed presence of CD20+ B-cells, CD3+ T-cells and PD-L1+ cells aggregated in immune cell foci known as tertiary lymphoid structures (TLS) in non-tumor region. (A) Representative AperioScope scanned image of anti-CD20 stained prostatectomy section counter-stained with hematoxylin. Tumor regions outlined by pathologist markings in blue, T = tumor, NT = non-tumor, 10× magnification. (B) Box inset enlarged at 200X magnification shows CD20+ B-cells stained brown in bright-field. (C) Post-deconvolution image of CD20 staining. After Imagescope deconvolution algorithm is run the stained color intensity is represented as image pixels with high intensity (brown), intermediate (orange) and low (yellow) staining intensity. Digital images of serial prostatectomy sections and de-convoluted images stained with anti-CD3 (D, E) and anti-PD-L1 (F, G).

## Data Availability

Individual participant data that underlie the results reported in this article, after de-identification (text, tables, figures, and appendices) will be available. The study protocol, statistical analysis, analytic code will be made available immediately after publication with no end date to researchers who provide a methodologically sound proposal. Proposals should be directed to camjamieson@health.ucsd.edu to gain access and data requestors will need to sign a data access agreement.
