# Supplementary figures and images for: Neoadjuvant rituximab modulates the tumor immune environment in patients with high risk prostate cancer
Source: J Transl Med. 2020 May 28;18:214. doi: 10.1186/s12967-020-02370-4 (PMC7257145; doi:10.1186/s12967-020-02370-4)

**Figure S1**


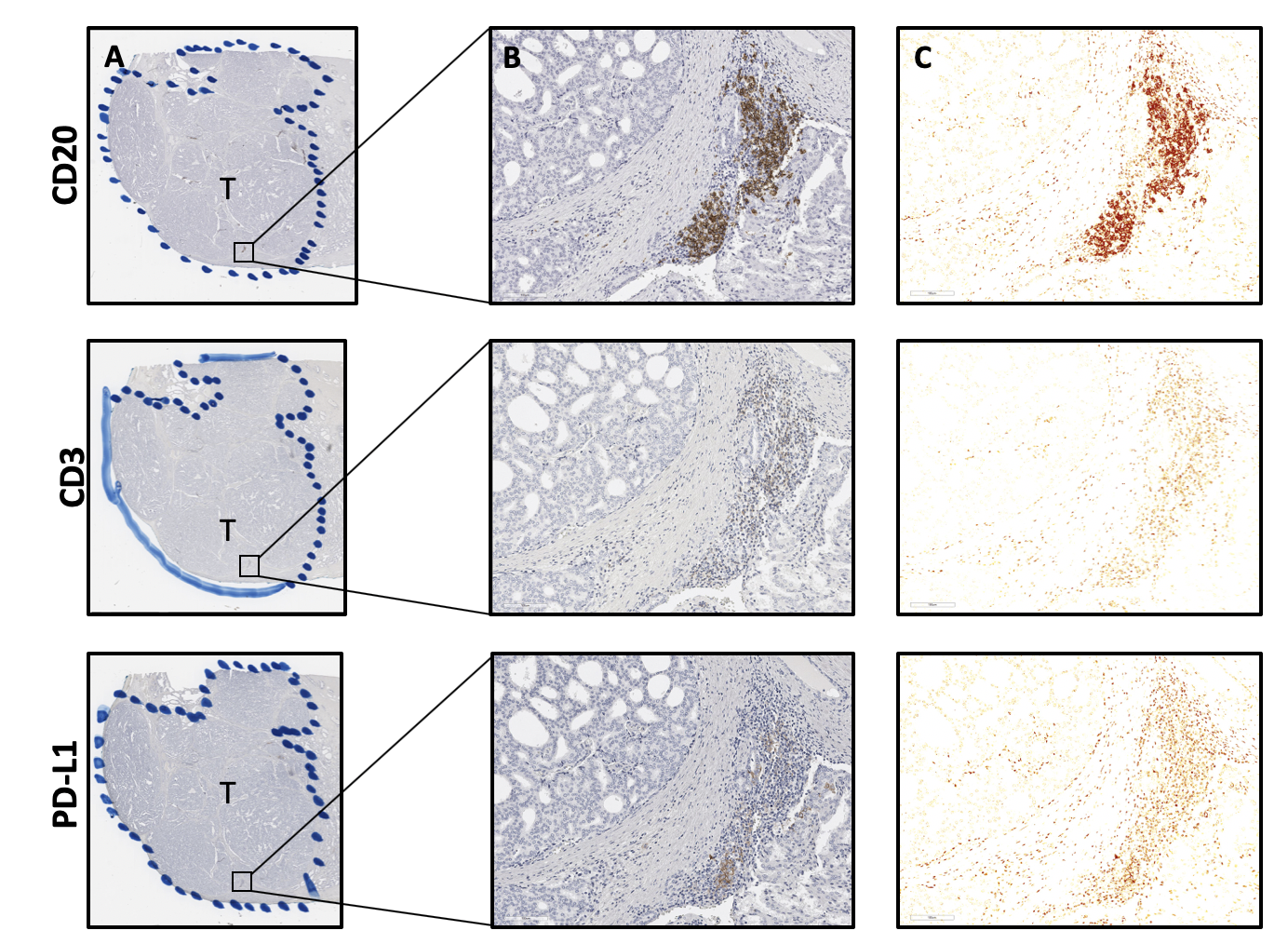


Figure S2


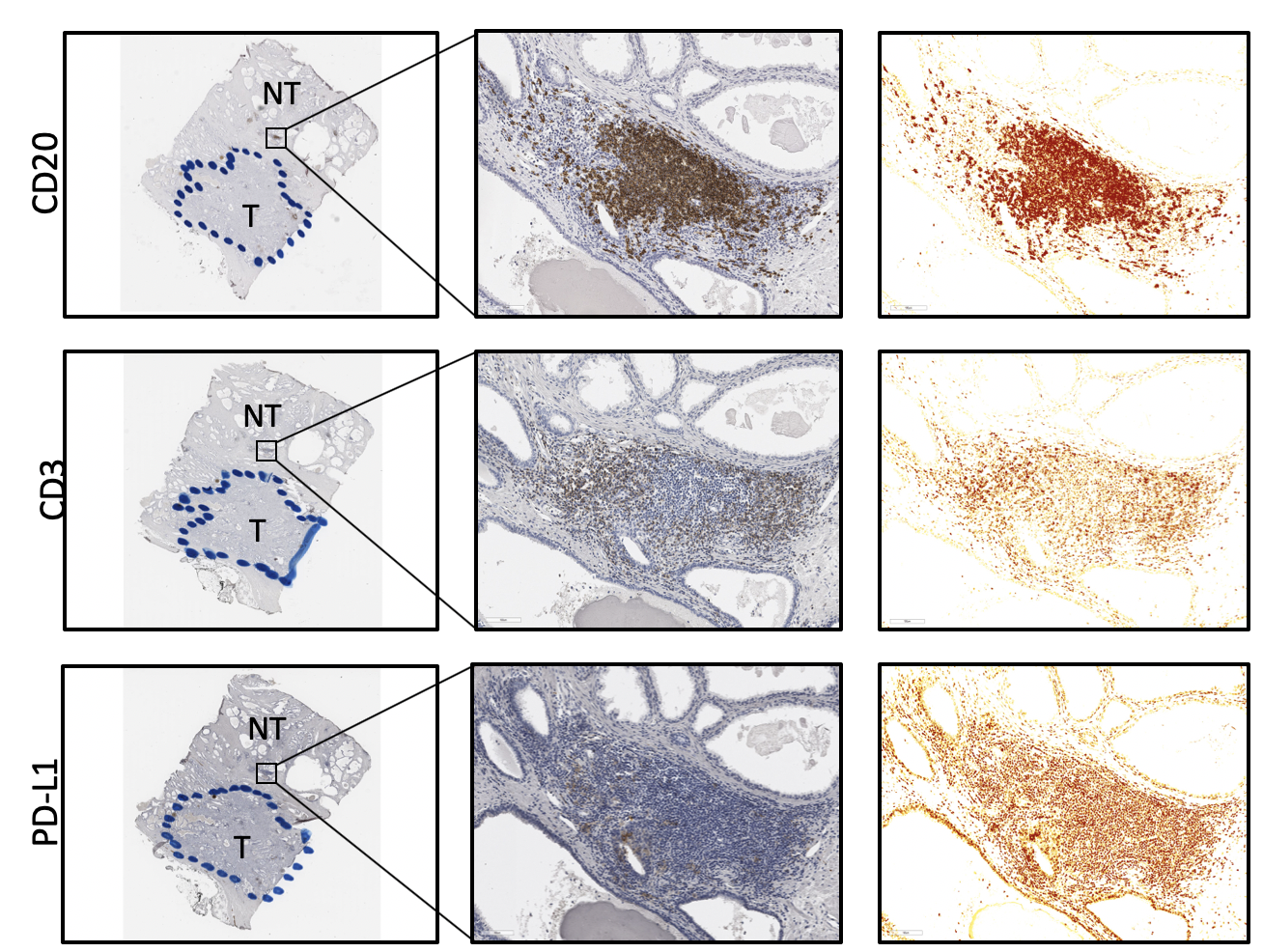

Supplement: Supplementary file 1 — Additional file 1: Figure S1. Immunohistochemical staining of serial prostatectomy sections showed presence of CD20+ B-cells, CD3+ T-cells and PD-L1+ cells aggregated in immune cell foci known as tertiary lymphoid structures (TLS) in tumor region. (A) Representative AperioScope scanned image of anti-CD20 stained prostatectomy section counter-stained with hematoxylin. Tumor regions outlined by pathologist markings in blue, T = tumor, NT = non-tumor, 10X magnification. (B) Box inset enlarged at 200× magnification shows CD20+ B-cells stained brown in bright-field. (C) Post-deconvolution image of CD20 staining. After Imagescope deconvolution algorithm is run the stained color intensity is represented as image pixels with high intensity (brown), intermediate (orange) and low (yellow) staining intensity. Digital images of serial prostatectomy sections and de-convoluted images stained with anti-CD3 (D, E) and anti-PD-L1 (F, G). Figure S2. Immunohistochemical staining of serial prostatectomy sections showed presence of CD20+ B-cells, CD3+ T-cells and PD-L1+ cells aggregated in immune cell foci known as tertiary lymphoid structures (TLS) in non-tumor region. (A) Representative AperioScope scanned image of anti-CD20 stained prostatectomy section counter-stained with hematoxylin. Tumor regions outlined by pathologist markings in blue, T = tumor, NT = non-tumor, 10× magnification. (B) Box inset enlarged at 200X magnification shows CD20+ B-cells stained brown in bright-field. (C) Post-deconvolution image of CD20 staining. After Imagescope deconvolution algorithm is run the stained color intensity is represented as image pixels with high intensity (brown), intermediate (orange) and low (yellow) staining intensity. Digital images of serial prostatectomy sections and de-convoluted images stained with anti-CD3 (D, E) and anti-PD-L1 (F, G). [file 12967_2020_2370_MOESM1_ESM.docx]
